# Orthodontics First in Hemimandibular Hyperplasia. “Mind the Gap”

**DOI:** 10.3390/ijerph17197087

**Published:** 2020-09-28

**Authors:** Stefania Perrotta, Giorgio Lo Giudice, Tecla Bocchino, Luigi Califano, Rosa Valletta

**Affiliations:** 1 Division of Orthodontics, Department of Neurosciences, Reproductive and Odontostomatological Sciences, University of Naples “Federico II”, 80138 Naples, Italy; perrottastefania@gmail.com (S.P.); teclabocchino2011@gmail.com (T.B.); rosa.valletta@unina.it (R.V.); 2 Maxillofacial Surgery Unit, Department of Neurosciences, Reproductive and Odontostomatological Sciences, University of Naples “Federico II”, 80138 Naples, Italy; luigi.califano@unina.it

**Keywords:** hemimandibular hyperplasia, skeletal asymmetry, mandibular asymmetry, condylar hyperplasia, orthognatic surgery, CAD/CAM

## Abstract

A 32-year-old man was referred to the Division of Orthodontics of the University of Naples “Federico II”, with a 15-year history of gradually increasing right-sided facial asymmetry. Clinical and radiological examinations was consistent to hemimandibular hyperplasia, a rare developmental asymmetry characterized by three-dimensional enlargement of one-half of the mandible. The standard surgical-orthodontic management was proposed to the patient. However, he refused to undergo bimaxillary orthognatic surgery. Therefore, a different treatment was proposed based on the orthodontic technique of pre-surgical decompensation and post-surgical refinement used in bimaxillary orthognatic surgery planning, and surgical intervention with a condylectomy. The dental arches were evenly levelled out with a multi-bracket treatment and then the condylectomy was performed. Orthodontic treatment continued with a levelling and torque control by 0.19 × 0.25 SS arch and physiotherapy. At the three-month follow-up, the patient showed anterior and posterior bite rebalancing, arch intercuspation recovery, and anterior open bite closure due to muscular self-rebalancing. The two-year follow-up showed regular mandibular dynamic, orthodontic appliances were removed, and the patient was instructed to wear retainer for the following months. The final result was aesthetically reasonable for the patient, although slight asymmetry of the chin persisted.

## 1. Introduction

Hemimandibular hyperplasia (HH) is a rare developmental asymmetry characterized by three-dimensional enlargement of one-half of the mandible. The hyperplastic side usually involves the condyle, condylar neck, ramus, and body, with the anomaly terminating abruptly at the symphysis. Epidemiological data suggest that HH can occur at any age and can keep increasing past the time of growth with a statistical prevalence for the female sex [1]. The etiology of HH is not clearly defined, potentially stretching from genetic factors to endocrine disorders, vascular pathologies, and traumas [2]. Obwegeser et al. investigated the potential pathogenesis of the disease differentiating hemimandibular elongation (characterized by elongation of the mandible body without increase in height) and HH on a clinical and a biological level, analyzing the growth factors of both condyles, hence differentiating it from the merely hyperplasia of the condyle [3,4]. Histological analyses of this the patients’ condyles usually report the bone to be thick and irregular, with an increase of woven bone volume, hypertrophy of cartilage, and increase of undifferentiated germinating mesenchymal cells and chondrocytes in the subchondral bone [5,6,7]. The clinical analysis shows an increased height and medial shift of the affected side of the mandible with shortening and lateral shifting of the contralateral side, elongation of the mandibular alveolar process, bowing down of the lower border with low running mandibular canal, and affected gonial angle rounded off in a more caudal position. Maxillary bone and sinuses are compensatory altered showing downward growth and elongation of the alveolar process on the affected side. Occlusal examination usually shows Class II/2 malocclusion, tilting of occlusal plane and lip commissure, and rotation of the occlusion in the vertical and axial planes [8,9]. Diagnosis can be determined by radiographical exams although clinical diagnosis is still the gold standard. Nuclear imaging such scintigraphy, single-photon emission computed tomography (SPECT), and positron emission tomography (PET) are able to provide helpful insights on the activity level of the disease [10,11,12]. Treatment planning in adult patients usually requires maxillo-mandibular orthognatic surgery, condylectomy if it is active on scintigraphy scans, reshaping of mandibular lower margin, and genioplasty [13]. Virtual Surgical Planning (VSP) and computer-aided design (CAD)/computer-aided manufacturing (CAM) software can be used in these complex cranio-maxillofacial reconstructions in order to increase accuracy, reduce operating room time, and increase patient satisfaction. With the use of CAD/CAM software, the team can make various measurements and changes to the patient’s 3D craniofacial skeletal anatomy and account for yaw, pitch, and roll. Using CAD software, the resections and reconstruction are virtually planned pointing specific osteotomy locations with precision to the 1/100th of a millimeter [14].

The aim of this study was to propose an alternative approach to HH, using the orthodontic technique of pre-surgical decompensation and post-surgical refinement borrowed from bimaxillary orthognatic surgery planning, and surgical intervention with a condylectomy exclusively.

## 2. Case Report

**Figure 1:**
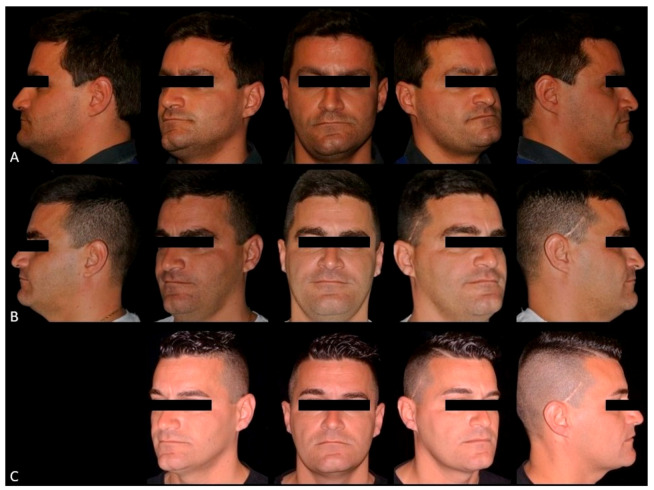
Extraoral view T0 (**A**); three-month post-surgical follow-up (**B**); two-year follow-up (**C**).

**Figure 2:**
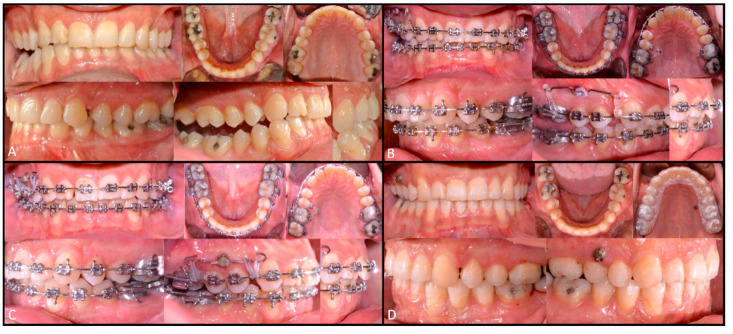
Intraoral view T0 (**A**); Pre-surgical (**B**); three-month post-surgical follow-up (**C**); two-year follow-up (**D**).

The study was conducted in accordance with the ethical principles of the Declaration of Helsinki (2002); approved by University of Naples “Federico II” Ethical Committee (n° 100/19), the patient gave consent for the publication of this paper. A 32-year-old man was referred to the Division of Orthodontics of the University of Naples “Federico II”, with a 15-year history of gradually increasing right-sided facial asymmetry. There was no history of trauma, infection, other systemic diseases, or facial surgery. T0 (baseline) extra-oral examination revealed significant facial asymmetry with left laterognathia, elongated right body and ramus of mandible and evident bascule of occlusal plane. No signs or symptoms of temporomandibular disorders were present. T0 intraoral examination revealed unilateral right open bite, left Class I malocclusion, right Class II malocclusion and slight shift of the mandibular midline toward the left side (Figure 1A and Figure 2A).

**Figure 3:**
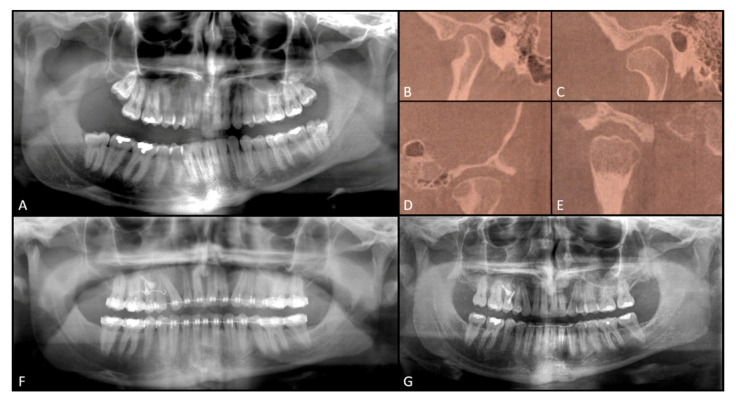
Radiographical analysis: T0 Orthopantomography (**A**); T0 Temporomandibular Joint (TMJ) Computed tomography scans of left TMJ in coronal (**B**) and sagittal view (**C**) and right TMJ in coronal (**D**) and sagittal view (**E**). Orthopantomography, three-week post-surgical follow-up (**F**). Orthopantomography, two-year follow-up (**G**).

Orthopantomography revealed significant enlargement of the mandibular condyle in the right side and comparatively normal condyle of the left side. The right gonial angle was characteristically bowing down and the mandibular canal was displaced to the lower right border of the mandible (Figure 3A).

**Figure 4:**
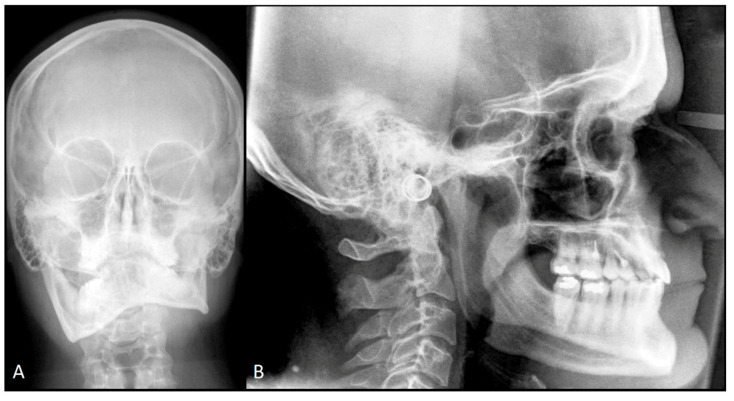
T0 skull radiography posteroanterior view (**A**); two-year follow up skull radiography laterolateral view (**B**).

Cephalometric analysis showed non-homothetic splits. Computed tomography and skull radiography was performed for further investigation, showing differences of size of both condylar heads as well as elongation of the neck of the mandibular right condyle (Figure 3B–E and Figure 4A).

**Figure 5:**
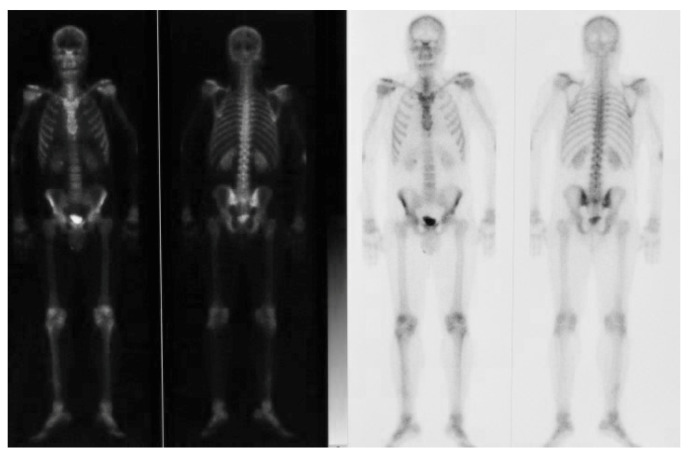
Total body scintigraphy showing no sites of pathological accumulation.

Bone scintigraphy was performed using a dose of 740 MBq of 99mTc-methylene diphosphonate and showed no hypermetabolic areas or pathological accumulation sites (Figure 5).

Clinical and radiographic findings were consistent with the diagnosis of hemimandibular hyperplasia of the right side. The standard surgical-orthodontic management was proposed to the patient; however, he refused to undergo the bimaxillary orthognatic surgery. Therefore, we proposed a different treatment, to whom he agreed.

The planning followed three steps:

- Decompensating orthodontic treatment
- Condylectomy
- Orthodontic refinement treatment

### 2.1. First Step

The dental arches were evenly levelled out with a multi-bracket treatment using 0.016 NiTi, 0.14 × 0.25 NiTi, and 0.19 × 0.25 NiTi arch sequence. Solid models of the mini-screws, teeth, and bone were imported and elaborated using ANSYS (ANSYS, v11.0, Swanson Analysis, Houston, PA, USA) to generate CAD models based on the screw insertion direction at the vestibular and palatal inter-radicular zones. To respect the CAD virtual planning, a customized surgical template was fabricated with a Ultra Violet (UV) Photocuring Liquid Crystal Display (LCD) 3D Printer (ELEGOO Mars Pro, Shenzhen Elegoo Technology Co, Shenzhen, China) with an offset of 1.8 mm thick, related to the mucosa and teeth model and according to the mini-screw insertion path. Whilst the levelling process continued, two mini-screws 8 mm and 10 mm long were inserted respectively on vestibular and palatal inter-radicular safe zones. The intrusion with temporary anchorage devices (TADs) of 3 mm of the right maxillary side was virtually planned using CAD/CAM software (Blue Sky Plan, Blue Sky Bio, Libertyville, IL, USA). Intrusive forces were applied on the right maxillary sector using elastic bands placed between a power arm installed on the vestibular mini-screw and the palatal one, changed and reactivated every three weeks. Ultimately, after 8 weeks, a 0.19 × 0.25 SS arch was installed in order to increase the right maxillary and mandibular arch gap up to 3 mm (Figure 2B). After two years from the beginning of the treatment, the patient was then addressed to the Maxillofacial Surgery Unit of the University of Naples “Federico II” for the surgery.

### 2.2. Second Step

**Figure 6:**
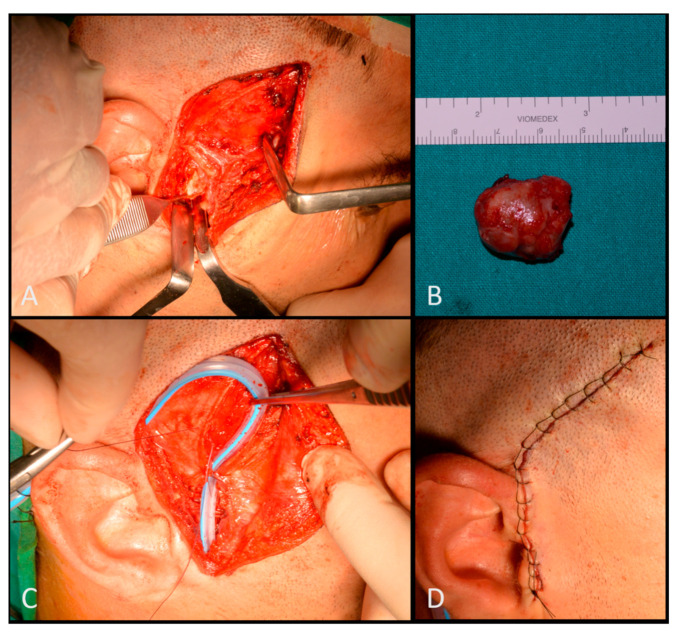
Intra-operatory view: Al-Kayat and Bramley preauricolar approach and TMJ exposure (**A**); condyle removal (**B**); Penrose drain positioning (**C**); and wound closure with running interlocking suture (**D**).

Under general anesthesia condylectomy was performed. The Al-Kayat and Bramley preauricular approach was chosen to maximize the exposure to the temporomandibular joint [15]. The condylectomy was performed by piezosurgery [16]. The Penrose drain was positioned. The wound closure was performed with running interlocking suture (Figure 6).

Aftercare had a regular course with no major complications. The immediate post-operative follow-up showed right side correct intercuspation, left and front side open bite, and overjet increase.

### 2.3. Third Step

**Figure 7:**
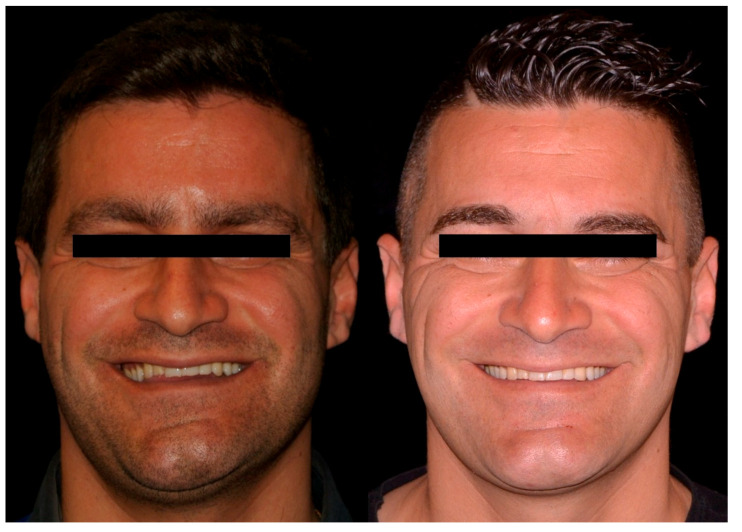
Maxillary occlusal cant correction from T0 (**left**) to two-year follow-up (**right**).

Orthodontic treatment continued with a levelling and torque control by 0.19 × 0.25 SS. After three weeks, the patient underwent physiotherapy to rehabilitate muscular tension on the new mouth opening pattern. At the three-month follow-up, the patient showed anterior and posterior bite rebalancing, arch intercuspation recovery, and anterior open bite closure due to muscular self-rebalancing (Figure 1B, Figure 2C and Figure 3F). The SS arch was again activated, and the power arm was modelled for canine distalization. The two-year follow-up showed regular mandibular dynamic, orthodontic appliances were removed, and the patient was instructed to wear retainer for the following months (Figure 1C, Figure 2D, Figure 3G and Figure 4B). Maxillary bascule was corrected thanks to the pre-surgical orthodontic intrusion (Figure 7).

## 3. Discussion

Patients affected by HH are usually treated, first orthodontically, using an arch progression sequence to reshape and realign the upper and lower dental arches. Then the surgical-orthodontic treatment should approach both maxillary bone and mandible, performing Le Fort 1 osteotomy with impaction on one side, condylectomy, sagittal split of mandibular rami, reshaping of mandibular lower margin, and genioplasty. Low condylectomy may be needed if the pathologic growth has expanded to the entire condylar volume. In young patients, a high condylectomy may be sufficient to control the abnormal growth pattern and normalize the development between the two sides of the mandible once the hyper-vascularized layer of the hyperplastic condyle is removed. In mild HH cases, a “wait and see” approach may be used until the cessation of condylar growth activity. However, care must be taken since the slow growing condyle may gradually affect the final facial and occlusal features after orthognatic surgery [17,18].

As soon as the patient refrained from the protocol proposed, the authors had to offer an alternative treatment that aimed to restore facial aesthetic and correct dental occlusion, respecting the patient’s will not to perform bimaxillary orthognatic surgery as recommended by standard treatment guidelines. This setback is usually linked due to side effects risks perception by the patient and lack of severity of functional chewing problem that could be managed with conservative treatments [19,20]. Hence, the surgical approach was limited to a right condylectomy and orthodontic treatment protocol had to be adjusted accordingly. The protocol proposal was based on the concept of decompensation used regularly for orthognatic surgery orthodontic treatment. Dentoalveolar compensation is a physiological phenomenon that occurs in most of severe occlusal discrepancies, capable of achieve normal inter-arch relationship [21]. Pre-surgical orthodontics in orthognatic surgery is required to contrast the physiological compensation, hence the definition as “decompensation”. Decompensation usually accentuates the patient’s malocclusion nonetheless allowing the surgeon to properly perform the bone segments movement and restore a normocclusion. In our case, we applied intrusive forces on sector 1 using elastic bands placed between two previously installed mini-screws. This procedure increased the inter-arch gap between sector 1 and sector 4, incrementing the right lateral open bite, but allowing a longer than usual upper translation of the frontal mandibular plane and the mandibular angle during the condylectomy. The right caudal occlusal plane canting was also orthodontically corrected, since surgically impacting the upper jaw on the right side was not an available choice. The intrusion movement was planned as 3 mm long during the pre-surgical stage. CAD/CAM software has been used in our case to plan the intrusion and the mandibular shift therefore guiding the condylectomy. The digital planning of all the operative phases allowed operators to perform a precise and minimally invasive treatment. The virtual pre-operative visualization of the area of interest should always start from a 3D Cone Beam Computed Tomography (CBCT) reconstruction. [22] From the elaboration of the DICOM files obtained, a CAD software along with a CAM device transform the surgical planning into a 3D printed guides, resulting into a more precise and predictable surgery. This versatile technology allows a multidisciplinary team to manufacture surgical splints, osteotomy cutting guides, and plate-bending templates via 3D printing that can be used for cranio-maxillofacial reconstructions, temporomandibular joint (TMJ) reconstructions, traumas, and oncological reconstructions. Correct mandibular kinetic was restored thanks to early physiotherapy treatment. While the chosen approach allowed us to reduce hospitalization time and reduce early post-operative distress common during bimaxillary orthognatic surgery due to edema and oral wounds, some limitations can be observed. Standard orthognatic surgery could have avoided possible further touch-ups for chin and mandibular lower margin recontouring. The patient in fact, lost weight over time, resulting in a sharper mandible profile that underlined the persisting small asymmetry of the chin in frontal view. Nonetheless, the result was aesthetically reasonable for the patient and coherent with his will of refusing additional surgeries. Relapse of the occlusal condition is unlikely to be expected since the growth activity of this hyperplasic mandible was assessed pre-operatively through bone scintigraphy that showed no hypermetabolic areas.

## 4. Conclusions

This report gives the opportunity to show how an orthodontic first approach to HH could be an alternative management to standard orthodontic-orthognatic treatment. Patients seeking for different and more conservative treatments may refuse standard surgical and orthodontic management of oral and maxillo facial pathologies. This digitally assisted treatment protocol could be used as guidance for dentists and surgeons looking for a balance between outcome results and patient satisfaction.

## References

[1] Raijmakers P.G., Karssemakers L.H., Tuinzing D.B. (2012). Female predominance and effect of gender on unilateral condylar hyperplasia: A review and meta-analysis. J. Oral Maxillofac. Surg..

[2] Almeida L.E., Zacharias J., Pierce S. (2015). Condylar hyperplasia: An updated review of the literature. Korean J. Orthod..

[3] Obwegeser H.L., Obwegeser J.A. (2010). New clinical-based evidence for the existence of 2 growth regulators in mandibular condyles: Hemimandibular elongation in hemifacial microsomia mandible. J. Craniofac. Surg..

[4] Obwegeser H.L., Makek M.S. (1986). Hemimandibular hyperplasia--hemimandibular elongation. J. Maxillofac. Surg..

[5] Gray R.J., Sloan P., Quayle A.A., Carter D.H. (1990). Histopathological and scintigraphic features of condylar hyperplasia. Int. J. Oral Maxillofac. Surg..

[6] Gray R.J., Horner K., Testa H.J., Lloyd J.J., Sloan P. (1994). Condylar hyperplasia: Correlation of histological and scintigraphic features. Dentomaxillofac. Radiol..

[7] Eslami B., Behnia H., Javadi H., Khiabani K.S., Saffar A.S. (2003). Histopathologic comparison of normal and hyperplastic condyles. Oral Surg. Oral Med. Oral Pathol. Oral Radiol. Endod..

[8] Bertolini F., Bianchi B., De Riu G., Di Blasio A., Sesenna E. (2001). Hemimandibular hyperplasia treated by early high condylectomy: A case report. Int. J. Adult Orthodon. Orthognath. Surg..

[9] Obwegeser H.L. (2001). Mandibular Growth Anomalies: Terminology, Aetiology, Diagnosis, Treatment.

[10] Saridin C.P., Raijmakers P.G., Tuinzing D.B., Becking A.G. (2011). Bone scintigraphy as a diagnostic method in unilateral hyperactivity of the mandibular condyles: A review and meta-analysis of the literature. Int. J. Oral Maxillofac. Surg..

[11] Lewis E.L., Dolwick M.F., Abramowicz S., Reeder S.L. (2008). Contemporary imaging of the temporomandibular joint. Dent. Clin. N. Am..

[12] Romano A., Lo Faro C., Salzano G., Dell’aversana Orabona G., Petrocelli M., Corvino R., Vaira L.A., Iaconetta G., Califano L. (2018). Synovial chondromatosis of temporomandibular joint spreading into the cranial space. J. Oral Maxillofac. Surg. Med. Pathol..

[13] Marchetti C., Cocchi R., Gentile L., Bianchi A. (2000). Hemimandibular hyperplasia: Treatment strategies. J. Craniofac. Surg..

[14] Wang Y.Y., Fan S., Zhang H.Q., Lin Z.Y., Ye J.T., Li J.S. (2016). Virtual Surgical Planning in Precise Maxillary Reconstruction With Vascularized Fibular Graft After Tumor Ablation. J. Oral Maxillofac. Surg..

[15] Al-Kayat A., Bramley P. (1979). A modified pre-auricular approach to the temporomandibular joint and malar arch. Br. J. Oral Surg..

[16] Lo Giudice R., Puleio F., Rizzo D., Alibrandi A., Lo Giudice G., Centofanti A., Fiorillo L., Di Mauro D., Nicita F. (2019). Comparative investigation of cutting devices on bone blocks: An SEM morphological analysis. Appl. Sci..

[17] Ferguson J.W. (2005). Definitive surgical correction of the deformity resulting from hemimandibular hyperplasia. J. Craniomaxillofac. Surg..

[18] Xu M., Chan F.C., Jin X., Xu J., Lu J., Zhang C., Teng L. (2014). Hemimandibular hyperplasia: Classification and treatment algorithm revisited. J. Craniofac. Surg..

[19] Hagensli N., Stenvik A., Espeland L. (2014). Patients offered orthognathic surgery: Why do many refrain from treatment?. J. Craniomaxillofac. Surg..

[20] Santagata M., De Luca R., Lo Giudice G., Troiano A., Lo Giudice G., Corvo G., Tartaro G. (2020). Arthrocentesis and sodium hyaluronate infiltration in temporomandibular disorders treatment. Clinical and MRI evaluation. J. Funct. Morphol. Kinesiol..

[21] Solow B. (1980). The dentoalveolar compensatory mechanism: Background and clinical implications. Br. J. Orthod..

[22] Lo Giudice R., Nicita F., Puleio F., Alibrandi A., Cervino G., Lizio A.S., Pantaleo G. (2018). Accuracy of periapical radiography and CBCT in endodontic evaluation. Int. J. Dent..

